# Detecting groups of coevolving positions in a molecule: a clustering approach

**DOI:** 10.1186/1471-2148-7-242

**Published:** 2007-11-30

**Authors:** Julien Dutheil, Nicolas Galtier

**Affiliations:** 1 Institut des Sciences de l'Évolution (UM2-CNRS) Université Montpellier 2 Place Eugéne Bataillon, CC064, 34 095 Montpellier cedex 5, France

## Abstract

**Background:**

Although the patterns of co-substitutions in RNA is now well characterized, detection of coevolving positions in proteins remains a difficult task. It has been recognized that the signal is typically weak, due to the fact that (i) amino-acid are characterized by various biochemical properties, so that distinct amino acids changes are not functionally equivalent, and (ii) a given mutation can be compensated by more than one mutation, at more than one position.

**Results:**

We present a new method based on phylogenetic substitution mapping. The two above-mentioned problems are addressed by (i) the introduction of a weighted mapping, which accounts for the biochemical effects (volume, polarity, charge) of amino-acid changes, (ii) the use of a clustering approach to detect groups of coevolving sites of virtually any size, and (iii) the distinction between biochemical compensation and other coevolutionary mechanisms. We apply this methodology to a previously studied data set of bacterial ribosomal RNA, and to three protein data sets (myoglobin of vertebrates, S-locus Receptor Kinase and Methionine Amino-Peptidase).

**Conclusion:**

We succeed in detecting groups of sites which significantly depart the null hypothesis of independence. Group sizes range from pairs to groups of size ≃ 10, depending on the substitution weights used. The structural and functional relevance of these groups of sites are assessed, and the various evolutionary processes potentially generating correlated substitution patterns are discussed.

## Background

Measuring the non-independence (= coevolution) of positions (= sites) within a molecule – particularly a protein – is a major challenge of molecular evolution, as witnesses the large methodological effort achieved during the last decade (reviewed in [1]). Several theoretical and experimental arguments link the non-independence of sites to structural and/or functional constraints in proteins. The mechanism most often invoked is the occurrence of compensating mutations, either locally or distantly. Local coevolution may reflect direct residue-residue interaction, whereas distal coevolution is supposed to be the result of more complex mechanisms like secondary structure shifts [2]. But coevolution may also be defined in a broader sense, as correlated evolution: two sites are said to be non-independent if they tend to undergo substitution events in the same branches of the tree. Such events are called co-substitutions [3].

The main pitfall in coevolution detection (and more generally in comparative analysis of biological data) is to distinguish the functional signal from the phylogenetic noise. The latter is due to the shared history of all sites, represented by the underlying phylogenetic tree. Several methods have been developed to assess the departure from independent evolution [2-12]. They differ by (i) the statistic used to measure non-independence (correlation, mutual information), and especially whether it explicitly accounts for the compensatory nature of mutations or not, (ii) the assessment of the level of statistical significance (analytical, randomization, simulation) and (iii) the way they deal with phylogenetic inertia [1]. Global evidence of non-independent evolution between sites has been reported. It has been shown that amino acids in close proximity (see for instance [7,12]) or belonging to the same proteic domain [8,13] tend to evolve in a more correlated way than randomly chosen sites. The specific task of pointing to non-independently evolving sites in a given data set, however, suffers from methodological fuzziness and/or deficient software implementation. This probably explains why coevolution detection methods are not routinely used in pipelines for genomic annotation.

Here we introduce a new methodology to detect coevolving groups of sites within a molecule, based on the evolutionary history of these sites. The process is inferred by probabilistic substitution mapping, as we presented in a previous work [11], and is now extended to the protein case. The mapping procedure provides, for each site, an estimate of the number of substitutions in each branch of the tree. This procedure is extended to account for the biochemical properties of the amino acids, by weighting substitution events according to the physico-chemical distance between amino acids. We then define coevolving sites either as sites showing correlated substitution histories, extending the approach of [3], or by sites exhibiting explicit compensation.

It is likely that in proteins one substitution can be compensated by several other substitutions occurring at different positions. Looking for pairs of coevolving sites, as attempted so far, may therefore underestimate the coevolution signal in proteins. Performing an exhaustive search of groups of arbitrary size, however, is inefficient, and in most cases impossible, due to the high number of possible combinations. Clustering techniques are standard methods designed to cope with this issue. Surprisingly, these methods have never been applied to the detection of coevolution. We present here a hierarchical clustering approach to detect good candidate groups of coevolving sites. A statistical procedure is introduced to evaluate the significance of candidate clusters through parametric bootstrap. Using four example data sets, each with its own specificity, we provide evidence that this methodology is successful in making robust predictions of non-independently evolving positions.

## Results

### Algorithm

Given a sequence alignment and a tree, we first define a statistic measuring coevolution for an arbitrary group of sites. Then we introduce a method aiming at seeking candidate coevolving groups by clustering sites according to the coevolution statistics. Finally, we develop a statistical test to assess the significance of candidate groups.

#### Defining a coevolution statistic

**Figure 1:**
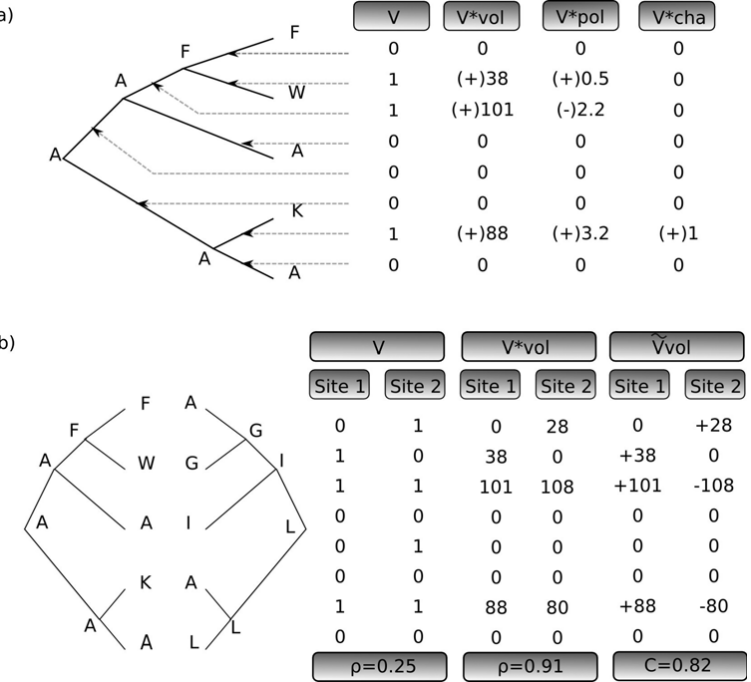
**Substitution mapping and measures of coevolution**. For the sake of simplicity, ancestral state uncertainty is not taken into account in this figure. True substitution vectors are averaged over all possible ancestral states. a) Unweighted (*V*), weighted (V*) and signed, weighted ($\tilde{V}$) substitution mapping, according to volume (vol), polarity (pol) and charge (cha). b) Coevolution measures for a pair of sites: correlation coefficient on the simple vectors, correlation coefficient on the volume-weighted unsigned vectors, and compensation index on the volume-weighted signed vectors.

Coevolution, as any evolutionary process, should be studied in the light of the phylogeny underlying the data, in order to distinguish functional correlations (resulting from convergence) from phylogenetic correlations (resulting from shared history). Mapping substitution events onto the phylogeny (substitution mapping) is a way to fully incorporate the evolutionary history of each site and has proved to be a powerful approach to infer coevolving positions [3,5,9,11]. Substitution mapping consists of estimating, for each site, the number of substitutions that occurred on each branch of the phylogenetic tree. These numbers are stored as a substitution vector for the site (noted *V*, Figure 1a), and can be computed using an empirical Bayesian approach (see methods and [11]). The site-specific, branch-specific numbers of substitutions depend on the set of ancestral states at inner nodes [3,11], the length of the branch and the rates of substitution between amino acids (or nucleotides). Since the ancestral states are inferred, we have to account for the uncertainty in the reconstruction, by averaging over all possible pairs of ancestral states. A fast, analytical procedure has been developed to achieve this calculation [11].

Here we introduce a generalization of this procedure called "weighted substitution mapping" (see Methods). It is dedicated to proteins and means weighting the different types of substitutions according to the resulting change in a given biochemical property of interest (Figure 1a). In the following, weighted substitution vectors are noted V*. Entry *k *of vector $V_{i}^{*}$ is then the estimated amount of biochemical change (*e.g*. change in volume, charge, polarity) having occurred at site *i *in branch *k*.

The amount of coevolution for a pair (*i*, *j*) of sites is measured by the correlation coefficient of the two substitution vectors (Figure 1b) [11]:

$$\rho_{ij}=\frac{cov(V_{i}^{*},V_{j}^{*})}{sd(V_{i}^{*})\times sd(V_{j}^{*})}.$$

If the two sites tend to undergo substitution events in the same branches, *ρ *will be positive and tend toward one. This measure is generalized to a group of arbitrary size *s *by defining the amount of coevolution for the group as the minimal pairwise correlation between sites in the group:

$$\rho =\underset{i,j\in 1..s}{\min}\{\rho_{ij}\}.$$

**Figure 2:**
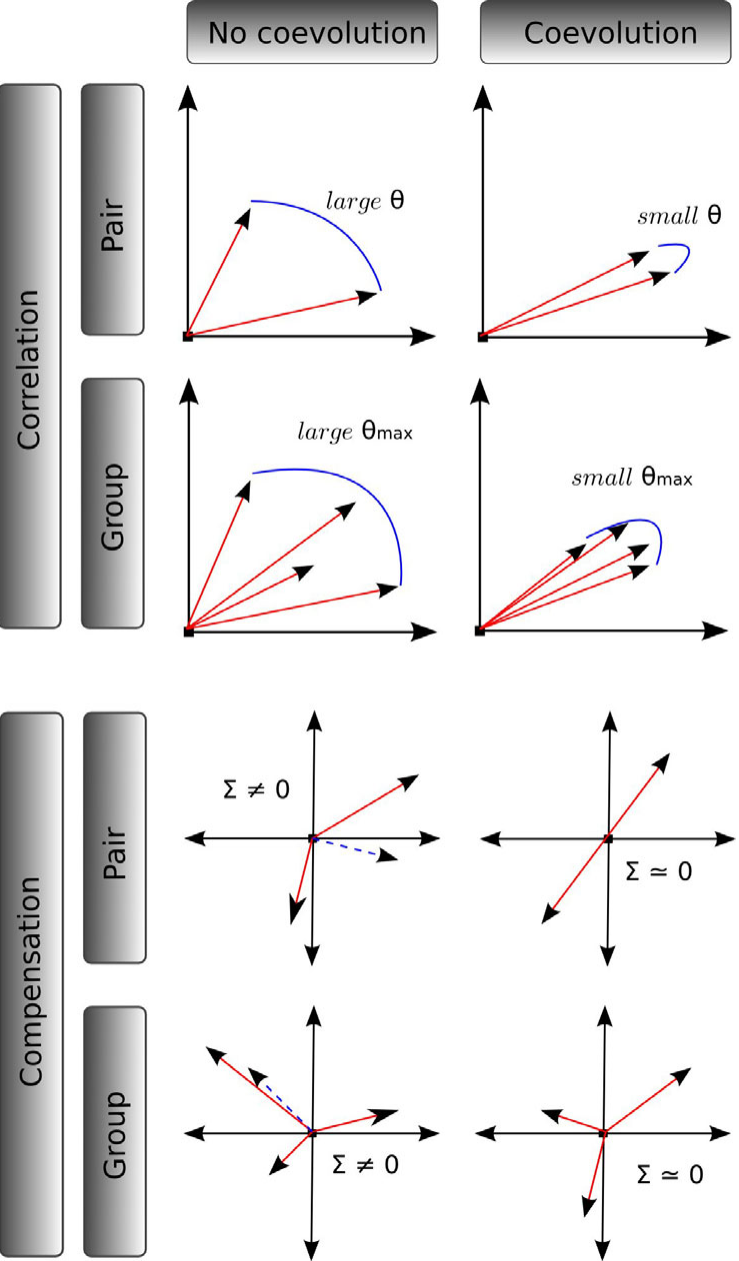
**Geometric interpretation of coevolution measures**. For simplification, vectors are plotted in a 2-coordinates space, which would correspond to a tree with only two branches.

From a geometrical point of view, the correlation coefficient is the cosine of the angle between the two substitution vectors, and the minimum correlation coefficient corresponds to the cosine of the maximum angle between the vectors of the group (Figure 2, upper panel).

The *ρ *statistic evaluates the tendency of sites to undergo correlated changes, irrespective of the compensatory nature of these changes. Weighted substitution mapping, however, offers the possibility to track the direction of changes, by giving opposite weight to *X *→ *Y *and *Y *→ *X *changes. The resulting signed, weighted vectors (noted $\tilde{V}$ in the following) can be used to test the compensatory nature of changes. The underlying assumption is that a given chemical property of a group of coevolving sites (global volume, charge, *etc*) would tend to be conserved, whereas the properties of individual sites may change. We hereby define *C*, the compensation index:

$$C_{ij}=1-\frac{\left|{\tilde{V}}_{i}+{\tilde{V}}_{j}\right|}{\left|{\tilde{V}}_{i}\right|+\left|{\tilde{V}}_{j}\right|},$$

where |*V*| is the length of vectors, that is the L2-norm:

$$\left|{\tilde{V}}_{i}\right|=\sqrt{\sum_{b}v_{ib}^{2}}.$$

In case of perfect compensation (the two vectors are symmetric), *C *equals 1. On the other extreme, if the vectors are identical, *C *equals 0. This measure can be generalized for a group of *s *sites:

$$C=1-\frac{\left|\sum_{i=1}^{s}{\tilde{V}}_{i}\right|}{\sum_{i=1}^{s}\left|{\tilde{V}}_{i}\right|}.$$

Compensation is high when the normalized length of the sum vector of all substitution vectors in the group (noted Σ in Figure 2, bottom) tends to $\vec{0}$. The correlation (*ρ*) and compensation (*C*) statistics therefore define two distinct detection methods, to be applied sequentially to a given data set.

As previously shown in several works, the interpretation to be given to coevolution statistics highly depends on the evolutionary rate of the sites under consideration [6,7,10,11]. Here we measured the variability of a site *i *by taking the length *N*_*i *_of its corresponding substitution vector (resp. $N_{i}^{*}$ for weighted substitution vectors):

$$\begin{array}{cc} N_{i}=\sqrt{\sum_{b}v_{ib}^{2}}, & N_{i}^{*}=\sqrt{\sum_{b}v_{ib}^{*2}} \end{array}$$

In the case of unweighted vectors accounting for multiple substitutions [11], this measure is proportional to the site-specific substitution rate. For weighted vectors, the N* statistics provides a new measure of the site-specific rate of change of a given biochemical property – note that the scale of N* depends on the weighting scheme used. The variability of a group of *s *sites was then defined as the minimum length of substitution vectors of all sites in the group, *Nmin*:

$$\begin{array}{cc} Nmin=\underset{i\in 1..s}{\min}\{N_{i}\}, & N^{*}min=\underset{i\in 1..s}{\min}\{N_{i}^{*}\}, \end{array}$$

which is a conservative measure since slowly evolving positions are the less informative. *Nmin *is a nuisance variable on which the coevolution statistics mechanically depend.

#### A clustering approach to select candidate groups

As the size of the tested groups augments, an exhaustive approach quickly becomes intractable, and heuristics should be developed. To detect groups (and not only pairs) of coevolving sites, we performed a cluster analysis of the substitution vectors. We used a complete linkage hierarchical clustering procedure, with the pairwise correlation and compensation distances defined as 1 - *ρ*_*ij *_and 1 - *C*_*ij *_respectively. The clustering procedure starts with the pairwise distance matrix *D*, and perform the following steps:

1. Find the pair (*u*, *v*) in *D *with the lowest distance, and cluster the two corresponding sites.

2. Remove the two selected sites *u *and *v *in the matrix, and add the new pair (*u*, *v*) as a single entry.

Compute all distances between the (*u*, *v*) pair and each remaining group using the formula:

*d*(*w*, (*u*, *v*)) = max(*d*(*w*, *u*), *d*(*w*, *v*))

3. Go back to 1, until the matrix size reaches 1.

The use of the maximum function defines the 'complete linkage'. Each step defines a new cluster, and reduces the matrix size by 1. Every cluster of the resulting bifurcating tree is considered as a candidate coevolving group of sites.

#### Assessing the significance of the amount of coevolution

The significance of the clusters was evaluated by parametric bootstrap. One thousand data sets with the same number of sites as the one of interest were simulated, using the estimated tree and parameters. The substitution vectors were computed and the clustering was performed. For each group in the resulting trees, we stored the size of the group, and the corresponding coevolution statistic (*ρ *or *C*) and *Nmin *values. We hence obtained, for each group size, the joint distribution of *ρ *(respectively *C*) and of *Nmin *under the null hypothesis of independence between sites. We then computed the p-value for a group of sites by conditioning over *Nmin*. For instance, for the coevolution statistic *ρ*:

p-value = Pr(*ρ *> *ρ*_*obs*_|*Nmin*_*obs*_)

where the *ρ*_*obs *_is the measured value for statistic *ρ*. Since *Nmin *is a continuous variable, we used a window centered on *Nmin*_*obs *_to evaluate the p-values:

$$\text{p-value}=\frac{N_{1}+1}{N_{2}+1}$$

where *N*_2 _is the number of simulation points with *Nmin *∈ [*Nmin*_*obs *_- *ω*/2, *Nmin*_*obs *_+ *ω*/2], and *N*_1 _is the number of simulation points in this range with a correlation greater or equal to the observed value. *ω *defines the size of the window. In this work we set it to 20% of the range of *Nmin *values.

One potential problem is the fact that nested clusters are not independent. Assume for instance that a triplet of sites actually coevolve. This should result in a significantly high *ρ *for the triplet itself, but perhaps also for pairs within the triplet, or even for certain *n*-uplets including the triplet, thus falsely duplicating the number of significant groups. To correct for this, the method outputs only one cluster – the one with the lowest p-value – when a series of nested clusters is detected. This is a conservative approach, and an improvement over pairwise methods, which typically tend to output large networks of residues (*i.e*. pair (*a*, *b*), pair (*b*, *c*), pair (*c*, *d*), *etc*, *e.g*. [10]).

#### Controlling the false discovery rate

Since we are performing repeated tests, we need to control for the global discovery rate. The clustering approach makes it impossible to rely on classical techniques, due to the non-independence of the tested groups. Here we assessed the false discovery rate by simulations: we conducted our analysis on several data sets simulated under the null hypothesis of independence. As an approximation, and to save computation time, we used the same simulated data sets as those used for computing the single groups p-values: one simulated data set was tested against the 999 remaining ones, and the p-values of the output clusters were recorded. This procedure was repeated 10 times. Now we pooled all the candidate clusters from the 10 analyses, and sort them by p-value. Then we sought the p-value threshold separating the 1% most significant clusters (those with lowest p-values) from the remaining 99%. This threshold is then applied to the real data set to ensure a 1% false discovery rate – only groups of the real-data analysis with a p-value lower than this threshold are considered significant after correction for multiple tests.

### Case studies

To assess its performance, the method was tested on four data sets, each with distinct properties. We now present the detailed results of these analyses and check the predictions with respect to substitution patterns and known protein structure and annotations.

#### Application to rRNA

We applied the new clustering approach with unweighted substitution mapping to a previously studied bacterial rRNA data set, containing 79 large subunit sequences with 2,312 sites [11]. Two hundred and sixty five clusters with a p-values lower than 1% were detected, containing 256 pairs. Two hundreds and forty nine of these pairs (97%) match already known structural stem pairs [14]. The pairwise approach [11] detected 258 pairs, among which 225 structural pairs (87%). The clustering approach hence has even lower false-positive rate than the pairwise approach. The power increase is probably due to the more elaborated p-value computation procedure. Higher order groups are distributed as this: four triplets, one quadruplets, two 5-uplets, one 6-uplets, and one 10-uplet. These groups may be of biological interest but their study is beyond the scope of this article. A detailed list of detected groups is provided as supplementary material.

#### Application to myoglobin

We applied the clustering approach to a myoglobin data set, which has been already scanned for coevolving pairs in previous works [3,6]. This data set contains 100 sequences, and 144 sites without gaps. In addition to the unweighted mapping, which ignores the nature of the substitutions, we tested four weighting schemes: volume, polarity and charge difference, and the synthetic Grantham chemical distance, a combination of volume, polarity and atom composition [15]. We ran both the correlation and the compensation tests (see methods).

The correlation test yielded 17 groups with a p-value lower than 5%, among which 13 remain significant after correction for multiple testing (see Table 1). Only two groups with a p-value lower than 1% were found (one group being detected by the "Grantham", "Volume" and "Polarity" method), indicating that the coevolution signal is weak in this data set, consistent with [6]. The compensation method leads to 21 groups at the 5% level (18 after correction for multiple testing), among which 10 were significant at the 1% level, and two at the 0.1% level (Table 2).

**Table 1:** Correlation analysis results for the myoglobin data set.

PDB	Size	Weight	Stat.	*Nmin*	p-value	FDR	3D dist.	3D p-value
THR39, GLY65, LEU76, GLU148	4	Volume	1	26.62	0.0044**	yes	28.21	0.1688†
THR39, GLY65, LEU76	3	Unweighted	1	0.98	0.0136*	yes	23.64	0.1409†
LEU11, ALA94, ILE111, ARG118, GLY150	5	Grantham	0.87	78.46	0.0045**	yes	38.14	0.7023†
LEU11, ALA94, ARG118, GLY150	4	Polarity	0.96	2.55	0.0065**	yes	38.14	0.8252†
LEU11, ALA94, ILE111, MET131, LEU135, LEU149, GLY150	7	Volume	0.82	20.89	0.007**	yes	33.1	0.1668†
GLY5, GLY23, GLU52, GLN91	4	Unweighted	0.47	2.67	0.0148*	yes	37.62	0.7952
LYS56, TYR103	2	Volume	0.96	84.01	0.0189*	yes	21.25	0.3506
ARG31, SER117	2	Volume	0.75	137.41	0.0241*	yes	11.89	0.023*‡
SER58, GLU85	2	Unweighted	0.72	1.9	0.0245*	yes	28.12	0.6853
ALA15, LYS63, ALA84, GLU85, GLN91	5	Grantham	0.5	133.99	0.032*	yes	27.7	0.0819.
ALA15, LEU61, ALA84	3	Unweighted	0.53	2.27	0.0346*	no	26.43	0.2817
ASP20, PHE33, LEU69, THR95	4	Volume	0.97	20.99	0.0347*	yes	31.15	0.3197†
PHE33, LEU69	2	Unweighted	1	1	0.0347*	no	16.14	0.1309‡
GLY121, ASP122	2	Unweighted	0.6	3.55	0.0379*	no	3.63	0.001***
ASP20, GLY23, ASP60, LYS63, ALA84, GLU85, LYS96	7	Charge	0.46	0.99	0.0379*	yes	29.33	0.035*†
GLU105, GLU136	2	Unweighted	0.74	1.77	0.0444*	no	8.25	0.003**
TRP7, LYS47, LYS62	3	Volume	0.71	87.27	0.045*	yes	35.94	0.8052

3D dist.: maximum pairwise 3D distance between alpha carbons (Å). 3D p-value: test if the sites are closer than expected by chance. FDR: tell if the group remains significant after a correction for having a global false discovery rate of 5%. ‡ symbols indicate groups detected by both the correlation and compensation analyses, † symbols indicate groups overlapping with a detected group in the compensation analysis (*i.e*. one group is a sub-group of the other).

**Table 2:** Compensation analysis results for the myoglobin data set.

PDB	Size	Weight	Stat.	*Nmin*	p-value	FDR	3D dist.	3D p-value
PHE33, LEU69	2	Volume	1	20.99	2e-04***	yes	16.14	0.1339‡
PHE33, LEU69	2	Grantham	1	21.99	3e-04***	yes	16.14	0.1339‡
PHE33, LEU69	2	Polarity	1	0.3	0.0016**	yes	16.14	0.1339‡
LEU11, LYS50, TYR103, ARG118	4	Polarity	0.83	5.63	0.0014**	yes	34.22	0.5534
ALA94, GLY150	2	Polarity	0.95	2.54	0.0027**	yes	8.78	0.006**†
ALA94, GLY150	2	Grantham	0.93	77.72	0.0074**	yes	8.78	0.006**†
PRO37, ALA130	2	Grantham	0.96	94.93	0.0034**	yes	27.81	0.6753
ALA94, ILE111	2	Volume	0.87	67.97	0.0079**	yes	21.15	0.3866†
THR39, GLY65	2	Volume	0.98	27.63	0.0091**	yes	14.29	0.0699.†
ARG31, SER117	2	Volume	0.63	137.41	0.0093**	yes	11.89	0.023*‡
ARG31, SER117	2	Grantham	0.57	201.2	0.0253*	yes	11.89	0.023*‡
LYS42, ASP60, LYS63, THR70	4	Charge	0.71	1.41	0.0112*	yes	20.31	0.008**
GLN26, LYS63, GLU85, TYR103	4	Grantham	0.76	132.11	0.0137*	yes	22.9	0.018*
ASP20, LYS96	2	Charge	0.6	2.23	0.0149*	yes	29.33	0.7353†
THR95, LYS145, GLU148	3	Polarity	0.63	7.42	0.0194*	yes	11.85	0.002**
LEU11, PHE138, GLY150	3	Volume	0.68	41.27	0.0333*	yes	33.1	0.6703
LYS78, GLU85, ILE99	3	Volume	0.68	37.79	0.0373*	yes	23.37	0.1369
VAL21, GLN26, ASP27, VAL66, ALA74, LYS78	6	Charge	0.72	1.41	0.0415*	no	24.79	0.006**
LEU9, GLU52, ALA53	3	Polarity	0.61	7.93	0.0415*	yes	33.3	0.6993
GLN8, LYS42, LYS50	3	Grantham	0.65	117.23	0.0455*	no	38.66	0.9171
GLY65, LEU76	2	Polarity	0.95	0.89	0.0475*	no	16.46	0.1449†

Legends are the same as in table 1.

**Figure 3:**
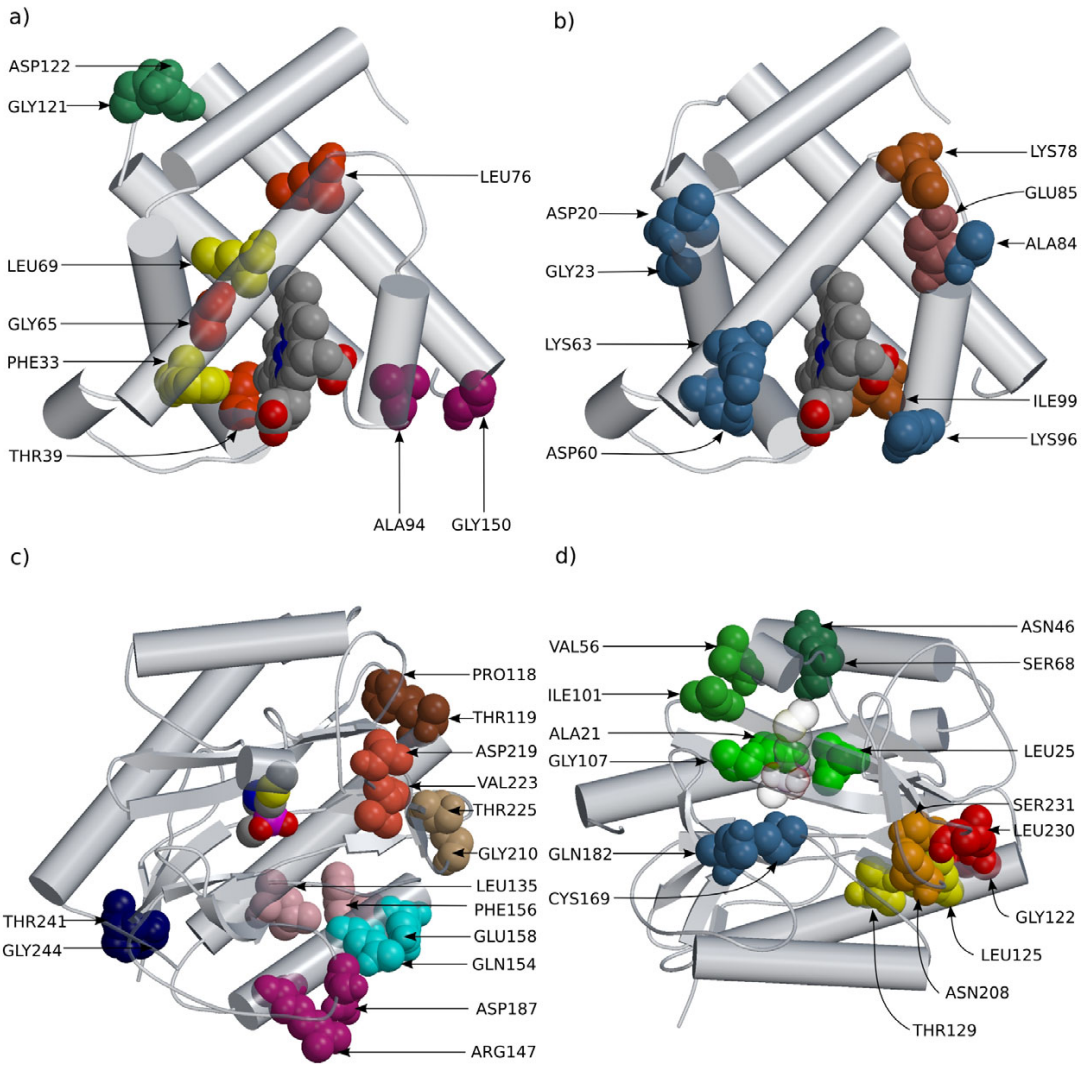
**Examples of detected groups**. a) and b): myoglobin, c) and d): Methionine Amino-Peptidase. All amino acids sharing the same color are detected as coevolving. Figures are made with the molscript [43] and raster3d programs [44], using PDB entries 1MBD for myoglobin, and 1C24 for MAP.

Detailed examination of the detected groups revealed interesting patterns. Several sites were in significantly close proximity (*e.g*. sites GLY121 and ASP122, located in a external loop of the molecule, ALA94 and GLY150 which are in the end of two helices (see Figure 2A), and sites ARG31 and SER117). Several sites (among the most significant ones) appeared to be located close to the heme group (LEU76, GLY65, THR39 and PHE33, LEU69, see Figure 3a). Finally, we noticed a tendency for sites in helix ends to coevolve (see Figure 3b), a trend already mentioned by [6] and [9].

#### Application to SRK

**Figure 4:**
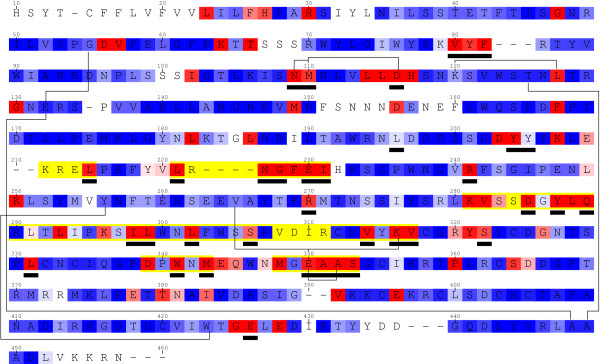
**A subset of the detected groups for the SRK data set**. Sequence of the SRK60 allele. Background colors indicate substitution rates as estimated by the Bayesian method [45], from blue (slow) to red (fast). Yellow boxes depict the hyper-variable regions, as defined by [46]. Black boxes underline positions reported to be under positive selection by [18]. Black lines show some of the most significant coevolving groups (see table 3) for detailed results.

The S-locus Receptor Kinase (SRK) is a molecule controlling self-incompatibility in various species of *Brassicaceae *[16]. The SRK gene is involved in pollen recognition, and is known to be under balancing selection, which results in trans-specific polymorphism [17]. The SRK protein is a transmembrane protein, with a cytoplasmic domain responsible for the kinase activity, and an ectodomain involved in receptor recognition. The ectodomain is highly polymorphic. It includes three hyper-variable regions (HVR), and several sites reported as undergoing positive selection [18] (see Figure 4). No three dimensional structure is available for this protein. The data set we used contains 53 sequences and 386 ungapped sites from the ectodomain.

**Figure 5:**
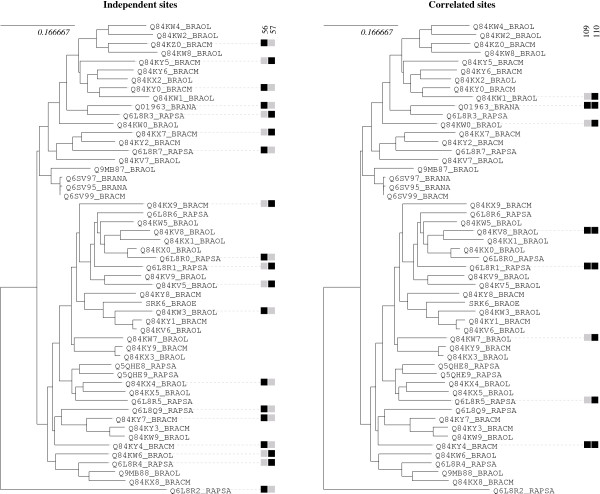
**Compared substitution maps for pairs (56, 57) and (109,110) of the SRK data set (see table 3)**. Black squares indicate an average number of substitutions greater than 0.9, gray squares indicate an average number lower than 0.9. Only nodes with at least one black square have been depicted for clarity. Two black squares on the same branch indicate a co-substitution event. The map on the left is for pair (56, 57), which is not detected as coevolving. The map on the right is for detected pair (109, 110), exhibiting several cosubstitution events.

The correlation test yielded 14 groups with a p-value lower than 5% after correction for multiple testing, among which 3 were significant at the 1% level (see Table 3 and Figure 4). The compensation test resulted in 24 significant groups at the 5% level, among which 23 remain significant after correction for multiple testing, and seven groups at the 1% level (see Table 4). The most significant group is a pair of sites (109, 110), detected using the unweighted mapping and the volume, polarity and Grantham weighted mappings with the correlation statistic. The corresponding unweighted mapping, shown in Figure 5, illustrates how correlated the substitution histories of these two sites have been. These sites had been reported as being under positive selection by Sainudiin *et al*. [18]. They are also detected with the compensation method and a Grantham weighting scheme, with a higher p-value. Site 110 is detected as coevolving with site 116 (also known to be under positive selection) for conserving a global polarity level. Another significant group of interest is the pair (350, 352), detected with the Grantham weighting, for the compensation test. These two sites are also reported to be under positive selection and are located in the second hyper-variable region. Together with the study of Sainudiin *et al*. [18], these results suggest a role for these sites in ligand recognition.

**Table 3:** Correlation analysis results for the SRK data set.

Alignment	Size	Weight	Stat.	*Nmin*	p-value	FDR
**109**, **110**	2	Unweighted	0.75	2.93	1e-04***	yes‡
**109**, **110**	2	Grantham	0.84	242.37	0.0033**	yes‡
**109**, **110**	2	Volume	0.79	154.32	0.0088**	yes‡
**109**, **110**	2	Polarity	0.76	8.82	0.0237*	yes‡
134, 186, 356, 383	4	Grantham	0.47	205.81	0.0115*	yes
54, 132, 394, 407, 448	5	Grantham	1	56	0.0121*	yes†
54, 55, 132, 248, 394, 407, 410, 429, 448	9	Polarity	0.97	0.7	0.0155*	yes†
54, 55, 132, 394, 407, 429, 448	7	Unweighted	0.71	1	0.0166*	yes†
130, 279	2	Charge	0.75	2	0.0232*	yes‡
120, 127	2	Charge	0.74	2	0.0251*	yes‡
25, 49, 70	3	Polarity	0.77	5.61	0.0278*	yes†
65, 121	2	Unweighted	0.66	2	0.0362*	yes
55, 132, 336, 387, 394, 432	6	Charge	1	1	0.0438*	yes†
265, **316**, **350**	3	Unweighted	0.45	2.22	0.0467*	yes

Legends are the same as in table 1. Sites in bold font are reported to be under positive selection by Sainudiin *et al*. [18].

**Table 4:** Compensation analysis results for the SRK data set.

Alignment	Size	Weight	Statistic	Nmin	p-value	FDR
125, 449	2	Grantham	1	57.93	0.0024 **	yes
125, 449	2	Volume	1	29.97	0.0028 **	yes
125, 449	2	Polarity	1	0.5	0.0047 **	yes
55, 132	2	Polarity	0.97	4	0.0039 **	yes†
132, 448	2	Grantham	0.98	107	0.0061 **	yes†
256, 423	2	Charge	1	1	0.0067 **	yes
120, 127	2	Charge	0.65	2	0.0099 **	yes‡
**350**, **352**	2	Grantham	0.54	314.34	0.0112 *	yes
130, 279	2	Charge	0.64	2	0.0118 *	yes‡
55, 336	2	Charge	1	1	0.0135 *	yes†
410, 448	2	Polarity	0.91	3.01	0.0152 *	yes†
25, 49	2	Polarity	0.83	5.61	0.0181 *	yes†
236, 375	2	Volume	0.89	62.99	0.0185 *	yes
23, 118	2	Grantham	0.84	141.51	0.0234 *	yes
23, 118	2	Volume	0.82	74.97	0.0288 *	yes
407, 433	2	Grantham	0.89	98.99	0.0256 *	yes
**110**, **116**	2	Polarity	0.54	12.87	0.0321 *	yes
394, 397	2	Charge	0.59	2	0.0338 *	yes
42, 49	2	Volume	0.73	104.95	0.0359 *	yes
96, 450	2	Charge	1	1	0.0369 *	yes
231, 347, 356	3	Polarity	0.61	10.05	0.0393 *	yes
**81**, **314**, 349, **353**	4	Volume	0.71	120.73	0.0473 *	yes
**109**, **110**	2	Grantham	0.53	240.8	0.048 *	yes‡
95, 278, 394, 433	4	Volume	0.77	28.94	0.0495 *	no

Legends are the same as in table 1.

The two tests detected sites that are conserved though all the alignment, except for the Q6L8R2_RAPSA allele. Sites 54, 394, 407, 132 and 448 for instance show a conserved polarity throughout the alignment, excepted for the Q6L8R2_RAPSA allele, which shows polarity change for the five sites. This allele is however very divergent from the other alleles (see Figure 3). It belongs to the so-called class II category, which contains only few alleles that are all recessive to alleles from the class I category [19]. These two categories form two distinct clades separated from each other about 40 millions years ago [20].

#### Application to MAP

**Figure 6:**
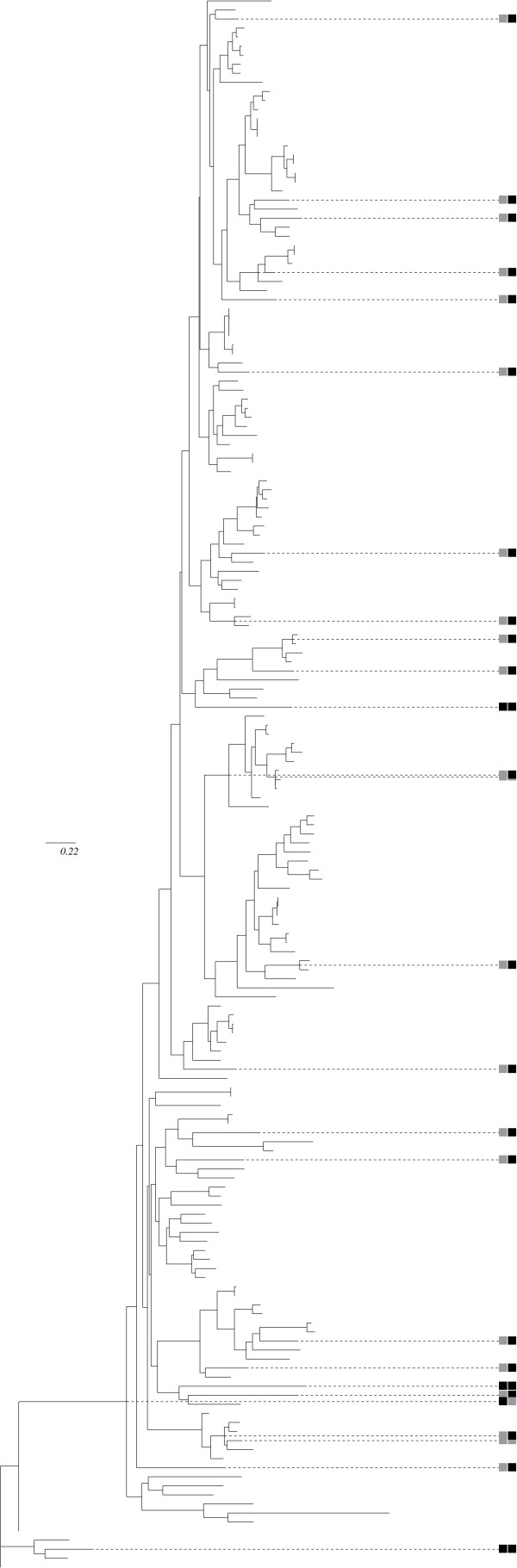
**Weighted and signed charge substitution mapping for pair (GLN154, GLU158) of the MAP data set (see table 6)**. Only mappings for branches with at least one site with at least ± 0.9 substitution on average have been represented for clarity. Black squares indicate positive change (- → +), and gray squares indicate negative changes (+ → -).

The last example of application is a data set previously studied for coevolution by Gloor *et al*. [10]. The coevolution signal was higher in this data set, probably at least partly because of the higher number of sequences (147), which leads to more powerful tests: a total of 43 groups with p-value < 1% were detected with the correlation test (among which 38 remain significant after correction for multiple testing). Surprisingly, the compensation statistic led to a lower number of detected groups (13, among which 10 remain significant after multiple testing correction). Most of the groups were pairs or triplets of sites in contact or in significantly close proximity (see Figure 3c and 3d). Several sites were also located in or close to the active site. Substitution maps for these groups clearly pointed to a large number of cosubstitution and compensatory events, as illustrated by Figure 6.

**Figure 7:**
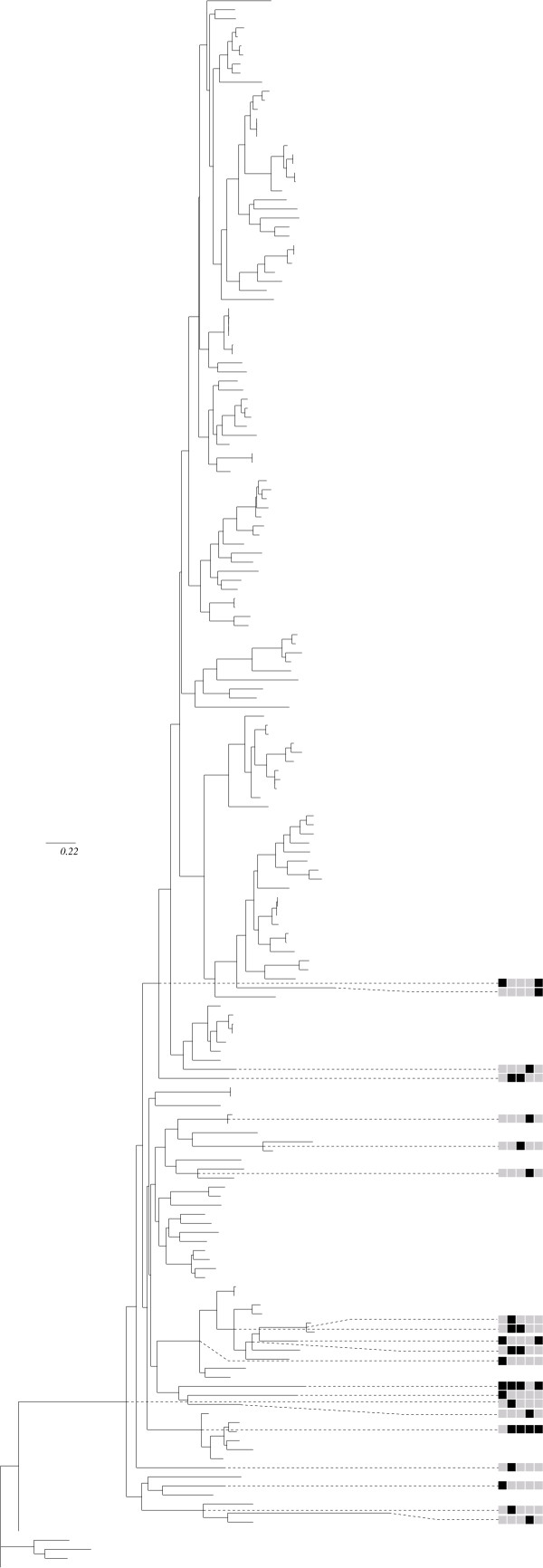
**Unweighted substitution mapping for pair (CYS78, ASN95, SER110, GLY150, GLN233) of the MAP data set (see tables 5), revealing a probable rate shift**. Only mappings for nodes with at least one site with at least ± 0.9 substitution on average have been represented for clarity. Same legends as in figure 3.

The most significant groups were also detected by Gloor *et al*. [10]. They found two kinds of coevolving pairs: one involving sites in close proximity, and a set of interconnected residues belonging to a large cluster. Gloor *et al*. [10] used mutual information (MI), after correcting for slowly evolving sites. Their statistic, however, does not account for the underlying phylogeny, which may lead to several false positives. In their study, this problem was addressed by considering only the most significant correlated sites, assuming that their correlation is above the background phylogenetic noise. By fully incorporating the phylogeny, we were able to confirm three of their predictions and add several new ones. These confirmed pairs are the three most significant ones according to our correlation method, and appear to be in contact in the three dimensional structure. A large proportion of our new predictions also appear to be close in the tertiary structure (see Tables 5 and 6, and Figure 3c and 3d). We were not able to confirm the most significant pair of Gloor *et al*. [10], neither pairs involved in the large network they report, with the exception of pair (CYS59, THR99) detected for volume compensation and which is close to the ligand. For some of the detected groups no obvious structural interpretation was found. In some cases, these apparent false positives corresponded to sites having undergone correlated changes of evolutionary rate. Figure 7 shows such an example. The five sites in Figure 7 are reasonably variable in general, but invariable in the top-most clade of the tree – presumably because they are under strong functional constraint in this group. Since changes are concentrated in a subset of the branches, the probability that these sites undergo co-substitutions by chance is higher than estimated using a model in which changes can occur throughout the tree, as in the simulations. This is a newly-reported mechanism by which correlated evolutionary patterns can appear in the absence of biochemical interaction between sites.

**Table 5:** Correlation analysis results for the MAP data set.

PDB	Size	Weight	Stat.	*Nmin*	p-value	FDR	3D dist.	3D p-value
ARG147, ASP187	2	Unweighted	0.57	4.92	0***	yes	6.24	0.001***
ARG147, ASP187	2	Grantham	0.59	517.54	0***	yes	6.24	0.001***
ARG147, ASP187	2	Volume	0.58	384.76	3e-04***	yes	6.24	0.001***
GLN154, GLU158	2	Unweighted	0.65	4.93	0***	yes	5.96	0.001***‡
GLN154, GLU158	2	Charge	0.67	4.1	1e-04***	yes	5.96	0.001***‡
GLY122, LEU230	2	Unweighted	0.6	6.42	1e-04***	yes	7.44	0.002**‡
GLY122, LEU230	2	Grantham	0.6	590.32	1e-04***	yes	7.44	0.002**‡
GLY122, LEU230	2	Polarity	0.61	18.34	1e-04***	yes	7.44	0.002**‡
GLY122, LEU230	2	Volume	0.48	275.16	0.0052**	yes	7.44	0.002**‡
ALA21, LEU25, GLY107	3	Unweighted	0.45	4.09	1e-04***	yes	12.07	0.001***
LEU25, GLY107	2	Grantham	0.52	312.81	0.0077**	no	12.07	0.031*
THR241, GLY244	2	Unweighted	0.57	3.24	3e-04***	yes	5.11	0.001***
ASN46, SER68	2	Unweighted	0.48	4.67	5e-04***	yes	5.92	0.001***
GLY210, THR225	2	Unweighted	0.56	3.25	5e-04***	yes	8.28	0.004**
ASP219, TRP221, THR222, THR225, ASP227, GLU235, ILE238, THR241, LEU248	9	Grantham	0.3	68.81	6e-04***	yes	40.12	0.2338†
TRP221, THR222	2	Unweighted	0.57	2.73	0.0014**	yes	3.75	0.001***†
TRP221, THR225,	3	Volume	0.56	105.03	0.0092**	no	18.13	0.024*†
ASP227								
LEU125, THR129	2	Unweighted	0.53	3.21	6e-04***	yes	6.12	0.001***
ASN208, SER231	2	Unweighted	0.48	4.32	7e-04***	yes	4.25	0.001***
TRP221, THR222, ALA232, GLU235, ILE238, VAL239, THR241, ILE247, LEU248	9	Polarity	0.28	2.15	0.001***	yes	34.73	0.025*
GLU148, GLU190	2	Unweighted	0.48	5.11	0.0011**	yes	11.88	0.03*
GLU148, GLU190	2	Polarity	0.5	13.91	0.006**	yes	11.88	0.03*
GLY210, GLY244, GLU246	3	Grantham	0.43	302.15	0.0012**	yes	33.45	0.4945
CYS78, ASN95, SER110, GLY150, GLN233	5	Unweighted	0.24	2.48	0.0018**	yes	21.37	0.001***
CYS169, GLN182	2	Unweighted	0.45	4.39	0.0018**	yes	6.4	0.002**
ILE96, TYR134	2	Unweighted	0.49	5.96	0.002**	yes	18.27	0.1768
ILE81, PHE113	2	Unweighted	0.45	4.33	0.0021**	yes	16.36	0.1259
ASP227, ALA232, GLU235	3	Unweighted	0.48	1.41	0.0022**	yes	23.49	0.0949.
ASP219, VAL223	2	Unweighted	0.46	3.66	0.0024**	yes	9.4	0.009**
HIS63, ARG127, GLN130, ALA209	4	Volume	0.33	205.12	0.0032**	yes	32.09	0.2527
HIS63, GLN130	2	Charge	0.47	5.36	0.0035**	yes	28.75	0.5994
THR222, SER231, ILE238, VAL239	4	Charge	0.71	0.99	0.0039**	yes	28.93	0.1199
ARG124, GLU131	2	Grantham	0.48	461.88	0.0043**	yes	10.6	0.016*
GLU131, VAL157, VAL164	3	Unweighted	0.36	4.39	0.0045**	yes	13.43	0.001***
GLN53, VAL98	2	Grantham	0.48	409.37	0.0049**	yes	13.51	0.0579.
GLU29, VAL32, ILE81, PRO82, THR129, ALA136, ILE149, GLY150, SER163	9	Polarity	0.21	0.1	0.005**	yes	32.31	0.008**
LEU135, PHE156	2	Grantham	0.49	575.88	0.0052**	yes	8.71	0.004**
GLN53, GLU160	2	Charge	0.44	5.85	0.0055**	yes	39.55	0.9311
VAL56, ILE101	2	Unweighted	0.51	6.53	0.0062**	yes	5.28	0.001***
VAL56, ILE101	2	Grantham	0.47	457.69	0.0069**	no	5.28	0.001***
LYS117, GLU123, ARG127	3	Grantham	0.4	487.3	0.0076**	no	15.43	0.004**
PRO118, THR119	2	Polarity	0.47	18.47	0.0096**	no	3.75	0.001***
ALA232, TYR234, GLU235	3	Charge	0.87	1.41	0.0098**	yes	10.51	0.002**

Legends are the same as in table 1. Only groups with a p-value lower than 1% are reported. A global false discovery rate (FDR) of 1% was used when correcting for multiple testing.

**Table 6:** Compensation analysis results for the MAP data set.

PDB	Size	Weight	Stat.	*Nmin*	p-value	FDR	3D dist.	3D p-value
CYS59, THR99, LYS141, SER231	4	Volume	0.68	147.26	3e-04***	yes	29.66	0.1289
TRP221, ASP227	2	Grantham	0.61	260.63	5e-04***	yes	18.13	0.1688†
LEU20, PRO34, ALA55, ASP90, SER132, VAL157, TRP221, ASP227	8	Polarity	0.75	9.96	8e-04***	yes	38.38	0.1548
GLY122, LEU230	2	Charge	0.49	3.78	9e-04***	yes	7.44	0.002**‡
HIS63, TYR134, GLU180, GLU190, PRO197, ASP242	6	Charge	0.7	3.89	0.0016**	yes	35.58	0.1678
GLN154, GLU158	2	Charge	0.48	4.09	0.0017**	yes	5.96	0.001***‡
PHE105, LYS111	2	Volume	0.47	267.57	0.0031**	yes	20.61	0.2458
VAL24, LYS67, ILE101	3	Volume	0.58	210.55	0.0033**	yes	15.15	0.002**
ASP227, HIS236	2	Charge	0.49	3.15	0.0046**	yes	27.22	0.5255
THR225, GLY244	2	Grantham	0.51	258.01	0.0064**	no	34.16	0.7802
VAL157, THR202	2	Grantham	0.47	310.7	0.0073**	no	18.42	0.1788
ARG19, GLU23, ASN46, VAL50	4	Charge	0.62	5.02	0.0086**	yes	16.04	0.001***
ALA17, ILE153, TRP221, THR222	4	Volume	0.66	126.09	0.0088**	no	26.58	0.0589.†

Legends are the same as in table 5.

It appears that the coevolution signal is weaker in proteins than in rRNA. Fewer groups are detected, with higher p-values and lower correlation statistics. These are groups for which changes occur more frequently in the same branches than expected by chance, but the linkage is not as strong as in rRNA.

### Implementation

The mapping procedure is available as dedicated classes in the Bio++ Phylogenetics library, from version 1.1 [21,22]. The coevolution detection can be reproduced using the CoMap program, including the pairwise analysis [11] and the clustering analysis (this article). For the clustering analysis, CoMap performs the clustering and the simulation part. As an option, it performs maximum likelihood estimation of model parameters prior to coevolution analysis, and supports a wide range of commonly used model of evolution, from Jukes-Cantor to General Time Reversible for nucleotides, and the Dayhoff and JTT models for proteins, using the dcmutt implementation [23]. CoMap can also read any user-defined model following the PAML format [24]. The p-value computation is done in R, using two scripts distributed along with the program. CoMap is open source and distributed at [25]. The rRNA and protein data sets are also available at this address, together with the results and the scripts used to run the analysis, which may serve as examples for running the coevolution analysis on other data sets. Running time, including the five types of mapping, was between two and five hours for the four data sets analyzed in this study, with a Intel(R) Xeon(TM) 3.06 GHz computer.

## Discussion

In this article we present a new test for detecting coevolving groups of sites based on weighted substitution mapping and a clustering approach. The method is original in (i) detecting groups of arbitrary size, (ii) accounting for the biochemical properties of amino-acid changes, (iii) distinguishing compensatory evolution from other kinds of correlated evolution, (iv) providing the substitution maps corresponding to the detected groups, so that the user can assess the relevance of the detected signal, and (v) being available as a user-friendly program with reasonable running times, easy to integrate into any sequence analysis pipeline.

When applied to a benchmark rRNA data set, the method recovered mostly pairs of sites, as expected, and was more powerful than the approach of [11], although the latter method had been specifically designed to detect pairs. When applied to protein data sets, the method detected candidate groups of coevolving sites of size two to ten. Many of these groups appear relevant from a structural or functional point of view. When applied to the MAP data set, the method detected a higher number of groups than the method of Gloor *et al.*[10], thanks to a more efficient phylogenetic control strategy, and direct assessment of the significance of groups of size higher than two. Coevolving residues were typically located in close proximity, or at the ends of helices, or corresponded to positively selected sites. The method also yielded coevolving groups of sites showing no obvious structural links. We now discuss in more details the various newly-introduced methodological improvements, and the biological mechanisms potentially underlying correlated evolutionary patterns.

### Weighted substitution mapping

A new probabilistic weighted substitution mapping was developed, thanks to the introduction of a weight matrix. A similar approach has been used by [8] and [26], without taking into account ancestral states reconstruction uncertainty. This mapping procedure can have some general interest beyond coevolution detection. It may converge toward the sub-alphabet approach (see for [3] and [18]), by using amino-acid categories (for instance big *versus *small, polar *versus *non-polar, charge *versus *not charges, *etc*) and defining the weight *w*_*x*,*y *_as equal to one if *x *and *y *do not belong to the same category, zero otherwise. Any other amino-acid index or distance may be used, as the ones available in the AAindex database [27]. The mapping is achieved using a fixed topology, assumed to be known. We have previously shown that the coevolution analysis is robust to variations of the underlying tree topology [11].

### Clustering

A usual pitfall when trying to detect coevolving sites without *a priori *knowledge is the high number of putative groups to be tested. An exhaustive approach is possible when only pairs of sites are targeted, but not for groups of arbitrary size: computing 2^*n *^- *n *- 1 p-values proves to be rapidly unfeasible. One possibility to overcome this issue is to limit the number of groups to test, thanks to the use of multivariate analysis (see [8]) or clustering techniques for instance. Here we used a hierarchical clustering approach, which outputs *n *- 1 clusters of size ≥ 2. A convenient property is that clusters of a same given size are independent and do not overlap. The clustering approach was useful for coevolution detection in drastically reducing the number of groups to be tested.

### Measuring cluster significance

We used two distinct measures of the amount of coevolution for a group. The first one, *ρ*, is the minimum pairwise correlation of sites within the group, and aims at detecting sites with correlated substitutions (co-substitution events). For a pair of sites, the *ρ *statistic is therefore equal to the correlation between the two (weighted) substitution vectors, a measure we introduced in a previous work [11]. The second statistic, *C*, explicitly accounts for the compensatory nature of substitutions. In a previous study, we showed that the coevolution statistic depends on the evolutionary rate of the sites, since slowly evolving sites tend to have artificially high correlation ([11], and also [7,9,13]). It is therefore necessary to include a measure of the site variability when assessing the significance of the correlation. We previously used the minimum posterior rate, a measure which is not easily generalizable to the case of weighted vectors. We hence used the length of substitution vectors as a measure of variability in this study. In the case of unweighted vectors, this measure is highly correlated to the posterior rate. To assess the variability of a group of any size, we used the minimum length of the corresponding substitution vector, *Nmin*. This is a summary statistic, since the variability of the group would be fully described only with the complete set of site-specific lengths. The *Nmin *measure is hence an approximation we employed to reduce the number of required simulations. Our approach may easily afford additional summary statistics in order to better account for group variability. We tried using both *Nmin *and *Nmax*. This did not affect the results, but removed some artificially detected groups of large size (> 10, results not shown). The results presented in this paper were obtained by using *Nmin *only and restricting the analysis to groups with a reasonable size, that is, lower than 10.

### Mechanisms of non independent evolution

In this work, coevolution has been defined in a broad sense, *i.e*. as equivalent to non-independent evolution. Sites are considered as coevolving when they tend to undergo (biochemically relevant) substitutions in the same branches of the tree. Co-evolution is hence seen as a non-random distribution of substitutions across the tree and alignment. This non-random distribution, however, may be due to distinct processes. The most intuitive scenario invokes compensatory mutations: one perturbing mutation at a site is compensated by one or several mutations at other sites to maintain a higher order structure. This mechanism corresponds to the definition of coevolution *sensu stricto*, used in most previous works. It relates to (intragenic) epistasis: the selection coefficient of a mutation at one site depends on the states at other sites. Correlated substitution does not necessarily imply compensation, or this compensation may not be seen at the single-molecule level. Sites involved in the recognition of an interacting partner, for instance, may tend to coevolve and be compensated by changes in the interacting molecule. More rarely, sites involved in adaptive process may also undergo correlated changes. Selection toward a new optimum requiring more than one substitution could explain the departure from independence [28]. This scenario should explain the signal detected in the SRK gene, where several coevolving sites have been previously reported to be under positive selection.

Another potential reason for non-independent evolution is a local relaxation of constraint involving several sites, the so-called heterotachy/covarion process [29,30]. If a structural or functional unit, generally required for proper functioning of the molecule, becomes useless in a specific lineage, then the sites forming this unit will accumulate changes in this lineage. This results in a correlation of rates, which may lead to correlated substitution mappings (see Figure 7). This correlation appears significant if a constant rate among sites is assumed when performing simulations. This may be corrected by using a covarion model as the one proposed by [31]. The two tests we introduce should help distinguishing between correlated states and correlated rates. In agreement with recent work by Hakes *et al*. [32], our analyses suggest that the former mechanism, neglected up to now, could explain a substantial fraction of the detectable correlated patterns.

## Conclusion

The flexible coevolution analysis we propose may provide powerful insight for understanding the evolutionary history of specific genes, similarly to positive selection detection analyses. Another open issue is the importance of coevolutionary processes in protein evolution in general [33]. Systematically applying our approach to a large number of data sets for which structural information is available could help making progress with this respect.

## Methods

The method analyses a set of aligned sequences (*D*) using a phylogeny (assumed to be known), a Markov substitution model and a discrete rate distribution across sites. The set of parameters Θ, including branch lengths, entries in the substitution matrix and rate distribution parameters are estimated using the maximum likelihood (ML) method prior to the co-substitution analysis.

### Substitution mapping

Let *D*_*i *_be the *i*^*th *^site of the data set, *i.e*. a column of the alignment. Let *v*_*i*,*b *_be the expected number of substitutions that occurred on branch *b *for site *i*. *v*_*i*,*b *_is computed as follow [11]:

$$v_{i,b}=\sum_{x_{p}}\sum_{x_{q}}\mathrm{Pr}(x_{p},x_{q}|D_{i},\Theta )\times n_{x_{p},x_{q}}(t)$$

where *x*_*p *_and *x*_*q *_are the states at the top and bottom node of the branch for this site. $n_{x_{p},x_{q}}(t)$ is the expected number of substitutions on a branch of length *t *knowing its initial state *x*_*p *_and final state *x*_*q*_. We previously showed that the exact computation of this number does not improve the coevolution detection [11], and may be approximated by taking:

$$n_{x_{p},x_{q}}(t)=\{\begin{array}{cc} 0 & \text{if }x_{p}=x_{q}\\ 1 & \text{if }x_{p}\neq x_{q} \end{array}$$

Equation 10 can be generalized to account for rate across site variation:

$$v_{i,b}=\sum_{c}\sum_{x_{p}}\sum_{x_{q}}\mathrm{Pr}(x_{p},x_{q},r_{c}|D_{i},\Theta )\times n_{x_{p},x_{q}}(t\times r_{c})$$

where the *c*th rate class has relative rate *r*_*c*_.

The first factor in the summation in equation 12 is the posterior probability of having state *x*_*p *_at bottom node, state *x*_*q *_at top node, and rate class *c *given the data and parameters. It can be computed using the formula:

$$\begin{array}{lll} \mathrm{Pr}(x_{p},x_{q},r_{c}|D_{i},\Theta ) & = & \frac{\mathrm{Pr}(x_{p},x_{q},r_{c},D_{i}|\Theta )}{\mathrm{Pr}(D_{i}|\Theta )}\\ & = & \frac{\mathrm{Pr}(x_{p},x_{q},D_{i}|\Theta ,r_{c})\times \mathrm{Pr}(r_{c})}{\mathrm{Pr}(D_{i}|\Theta )} \end{array}$$

where the first factor of the numerator is the likelihood for site *i *conditional on states *x*_*p *_and *x*_*q *_at top and bottom nodes and rate equal to *r*_*c*_. This likelihood is computed as described by Felsenstein [34], after having multiplied all branch lengths by *r*_*c *_[35] and summing over all possible ancestral states at each node except for the top and bottom nodes of branch *b*, for which states *x*_*p *_and *x*_*q *_are fixed. *P *(*r*_*c*_) is the prior probability for site *i *of being in rate class *c*, and *P *(*D*_*i*_|Θ) is the likelihood for site *i*. We call *V*_*i *_= (*v*_*i*,1_, ..., *v*_*i*,*b*_, ..., *v*_*i*,*m*_) (where *m *is the total number of branches in the tree) the *substitution vector *for site *i*.

This mapping can be applied to any alphabet type, including nucleotide and protein alphabets. We extended this procedure to account for the biochemical properties of amino-acid residues. We call $V_{i}^{*}=\left(v_{i,1}^{*},...,v_{i,b}^{*},...,v_{i,m}^{*}\right)$ the weighted substitution vectors, with

$$v_{i,b}^{*}=\sum_{c}\sum_{x_{p}}\sum_{x_{q}}\mathrm{Pr}(x_{p},x_{q},r_{c}|D_{i},\Theta )\times w_{x_{p},x_{q}}$$

where *W *= {*w*_*x*,*y*_} is a matrix of substitution weights.

We used the following weights, retrieved from the AAindex database [27]:

• Grantham physico-chemical distance [15], AAindex id: aax2:GRAR740104;

• Difference of volume (as defined by Grantham), AAindex id: aax1:GRAR740103;

• Difference of polarity (as defined by Grantham), AAindex id: aax1:GRAR740102;

• Difference of charge (K and R have +1, D and E have -1, 0 for the others.), AAindex id: aax1:FAUJ880111 and aax1:FAUJ880112;

These weights may be signed or unsigned. By setting *w*_*x*,*y *_= *w*_*y*,*x*_, we aim at detecting sites undergoing simultaneous changes in the given property (*i.e*. correlated evolution). Setting *w*_*x*,*y *_= -*w*_*y*,*x *_accounts for the direction of change, and may hence be used to detect compensatory changes. Signed substitution vectors are noted $\tilde{V}$. The four kinds of vectors were referred as "Grantham", "Volume", "Polarity" and "Charge" in this article. The unweighted vectors (equation 12) were also computed.

### Testing structure proximity

To assess whether sites of a coevolving group were in close proximity in a three dimensional structure, we measured the maximum *C*_*α*_-to-*C*_*α *_distance between residues in the group. We constrained the minimal primary distance between sites in randomized groups to be higher than five angstroms, or higher than the minimal primary distance in the tested group, when the latter was lower than five angstroms. We evaluated the significance of the obtained distance by randomization. One thousand groups of the same size were randomly picked from the structure, and their maximum distance measured. The p-value was calculated as the proportion of random groups showing a shorter maximal distance than the tested group.

### Data sets

To compare the clustering approach to the pairwise analysis, we re-analyzed a previously published 79 species bacterial LSU data set [11]. The method was then applied to three protein data sets: the myoglobin of vertebrates, the S-locus Receptor Kinase (SRK) gene of *Brassicaceae*, involved in sporophytic auto-incompatibility, and the Methionine Amino-Peptidase (MAP).

A hundred sequences of myoglobin were retrieved from the Swissprot database and aligned with ClustalX [36]. A phylogenetic tree was built with the PhyML program [37]. The topology was corrected by hand to match well-known branching orders, particularly within mammals [38]. Numerical parameters were then re-estimated while keeping the tree topology fixed.

SRK sequences were retrieved from Swissprot and trEMBL using SRS. Sequences with less than 100 amino acids were removed. A first alignment was performed using the Muscle program [39]. The alignment was visually inspected; ambiguously aligned sequences were removed. The ClustalX program was finally applied to the resulting data set. The final alignment included 53 sequences corresponding to the extra-cellular domain of the protein. A PhyML tree was constructed.

A data set of 174 aligned protein sequences of the Methionine Amino-Peptidase (MAP) was taken from [10]. Their structure-based alignment was used, and a phylogenetic tree was estimated using PhyML. For rRNA data, we used the HKY85 + Γ [40] substitution model, and for protein data the JTT92 + Γ model [41], implemented using the dcmut method [23]. A four-class discretized gamma rate distribution [42] was used in both cases. These models were used for tree building, substitution mapping and parametric bootstrap simulations.

## Availability and requirements

Project name: comap

Project homepage: 

Operating systems: Unix, Linux, MacOS and Windows.

Programming languages: C++, R

Other requirements: Bio++ libraries

License: CeCILL Free Software License (GNU GPL compatible, )

## Authors' contributions

NG and JD designed the method and wrote the manuscript. JD implemented the method and performed the analyses.

## Supplementary Material

Additional file 1 **Correlation analysis detailed results for the myoglobin data set, as an Open Document spreadsheet file**. 3D distance: maximum pairwise 3D distance between alpha carbons (A). Primary distance: °C distance, in amino acids, of the two positions on the protein sequence. 3D p-value: test if the sites are closer than expected by chance. FDR: tell if the group remains significant after a correction for having a global false discovery rate of 5%. ‡ symbols indicate groups detected by both the correlation and compensation analyses, † symbols indicate groups overlapping with a detected group in the compensation analysis (*i.e*. one group is a sub-group of the other). Click here for file

Additional file 2 **Compensation analysis detailed results for the myoglobin data set**. Legends are the same as in additional file 1. Click here for file

Additional file 3 **Correlation analysis detailed results for the SRK data set**. Legends are the same as in additional file 1. Sites in bold font are reported to be under positive selection by Sainudiin *et al*. [18]. Click here for file

Additional file 4 **Compensation analysis detailed results for the SRK data set**. Legends are the same as in additional file 1. Click here for file

Additional file 5 **Correlation analysis detailed results for the MAP data set**. Legends are the same as in additional file 1. Only groups with a p-value lower than 1% are reported. A global false discovery rate (FDR) of 1% was used when correcting for multiple testing. Click here for file

Additional file 6 **Compensation analysis detailed results for the MAP data set**. Legends are the same as in additional file 5. Click here for file
